# Methicillin-resistant *Staphylococcus aureus* meningitis following revision microdiscectomy: a case report

**DOI:** 10.1093/jscr/rjag868

**Published:** 2026-09-29

**Authors:** Zachary Troiani, Meaghan Gleason, David Kowalski, Ellen Lutnick, Maxwell Scott, Joseph Kowalski

**Affiliations:** Department of Orthopedic Surgery, University of Buffalo, 462 Grider St., Buffalo, NY 14215, United States; Jacobs School of Medicine and Biomedical Sciences, 955 Main St., Buffalo, NY 14203, United States; Department of Orthopedic Surgery, University of Buffalo, 462 Grider St., Buffalo, NY 14215, United States; Department of Orthopedic Surgery, University of Buffalo, 462 Grider St., Buffalo, NY 14215, United States; Department of Orthopedic Surgery, University of Buffalo, 462 Grider St., Buffalo, NY 14215, United States; Department of Orthopedic Surgery, University of Buffalo, 462 Grider St., Buffalo, NY 14215, United States

**Keywords:** MRSA meningitis, microdiscectomy, postoperative infection, spinal surgery complications

## Abstract

Healthcare-associated bacterial meningitis is a rare but life-threatening complication of spinal surgery, most commonly associated with incidental durotomy. A 59-year-old man developed fever, altered mental status, and generalized seizures 1week after uncomplicated revision L4–L5 microdiscectomy. Magnetic resonance imaging demonstrated diffuse intracranial and spinal leptomeningeal enhancement with a postoperative lumbar fluid collection. Cerebrospinal fluid (CSF) analysis was consistent with bacterial meningitis. Emergent irrigation and debridement revealed copious purulent fluid without evidence of a CSF leak despite meticulous dural exploration. Wound and CSF cultures grew methicillin-resistant *Staphylococcus aureus* (MRSA). Following surgical debridement and targeted intravenous vancomycin, the patient achieved substantial neurological recovery and was independently ambulatory with only mild residual cognitive impairment at 6 months. MRSA meningitis may occur after revision lumbar microdiscectomy without a recognized dural tear. Early recognition, surgical source control, and targeted antimicrobial therapy are essential to optimize outcomes.

## Introduction

Healthcare-associated bacterial meningitis is a rare but potentially devastating complication of spinal surgery, with an estimated incidence of 0.1%–0.3%. It most commonly occurs following incidental durotomy resulting in contamination of the cerebrospinal fluid (CSF), although cases have been reported without an identifiable dural injury [1–4]. Prompt diagnosis is essential because delayed treatment is associated with substantial morbidity and mortality.

Methicillin-resistant *Staphylococcus aureus* (MRSA) is a common cause of postoperative spinal infections and occurs more frequently after revision procedures than primary operations [5, 6]. However, MRSA meningitis following revision lumbar microdiscectomy has rarely been reported. We present a case of postoperative MRSA meningitis after revision lumbar microdiscectomy without an identifiable CSF leak, highlighting the diagnostic challenges, management, and favorable neurologic outcomes following timely intervention.

## Case report

A 59-year-old man underwent revision L4–L5 microdiscectomy for recurrent lumbar disc herniation 6 weeks after an index microdiscectomy. One week postoperatively, he developed worsening low back pain, chills, and progressive confusion, prompting presentation to the emergency department.

Initial examination demonstrated altered mental status and 1/5 strength in ankle dorsiflexion and great toe extension. Laboratory evaluation revealed mild leukocytosis and elevated inflammatory markers. Within a few hours after presentation, his neurologic status rapidly deteriorated, culminating in generalized seizures requiring endotracheal intubation and intensive care admission. Magnetic resonance imaging (MRI) of the brain and spine demonstrated a postoperative lumbar fluid collection with diffuse intracranial and spinal leptomeningeal enhancement, concerning for meningitis (Figs 1 and 2).

**Figure 1:**
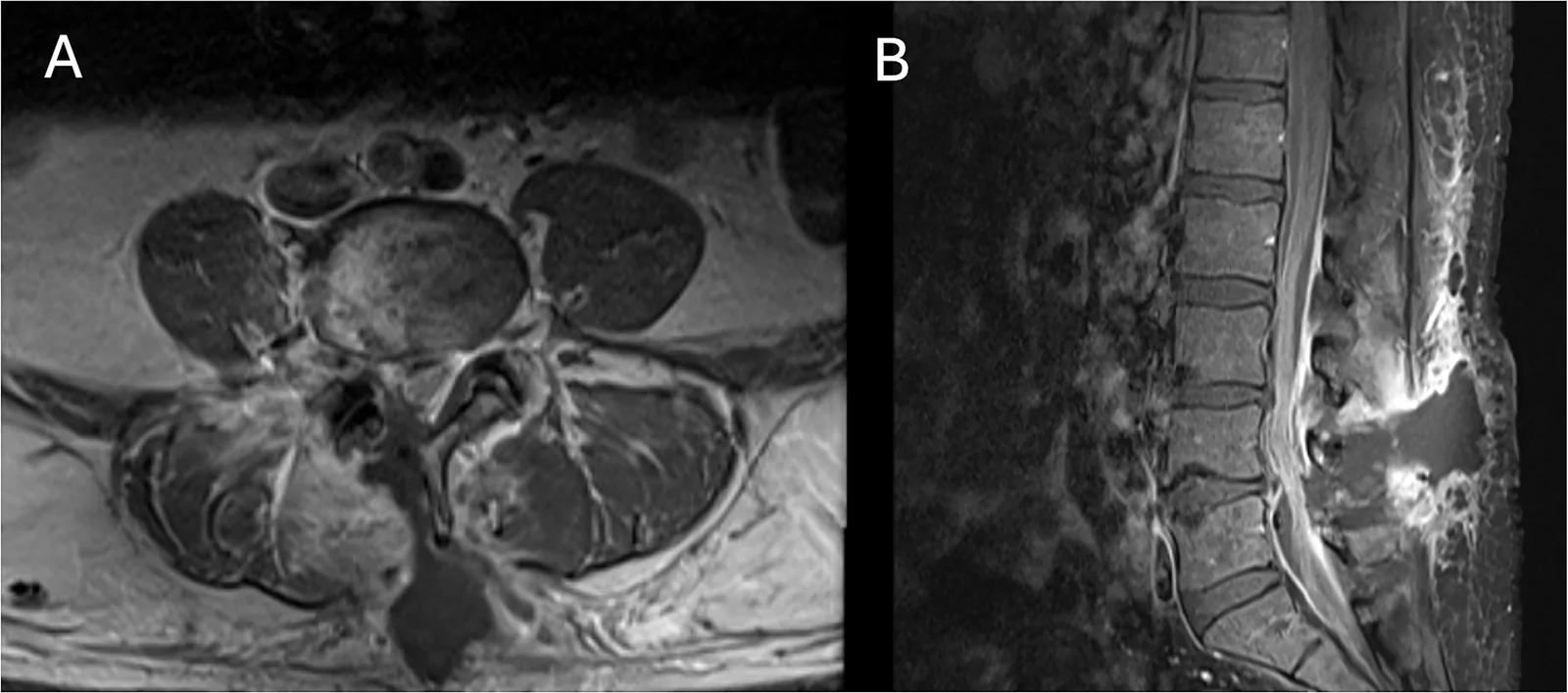
L4–L5 midaxial (A) and midsagittal (B) T1 postcontrast MRI demonstrating postop sequelae of decompressive right hemilaminectomy at L4–L5 with a fluid collection in the laminectomy site with adjacent enhancement of paraspinal musculature; there is trace subdural fluid collection along the posterior thecal sac and enhancement of the dura from L2-S1.

**Figure 2:**
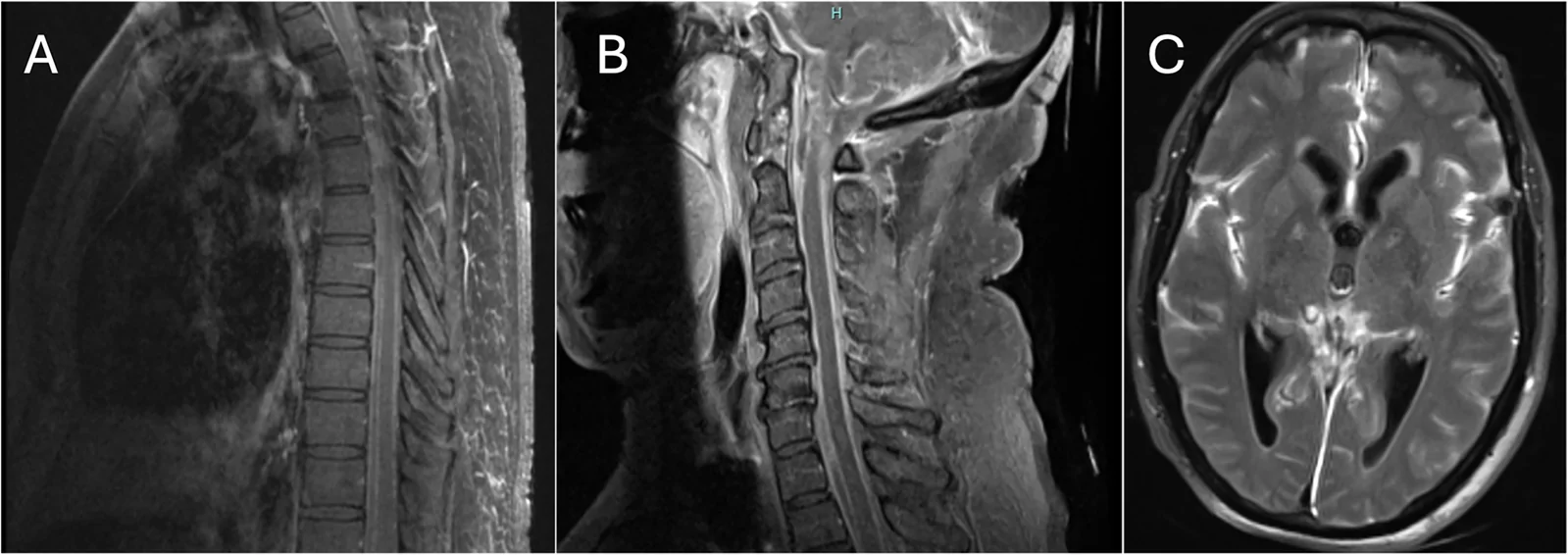
Thoracic (A) and cervical (B) midsagittal T1 postcontrast MRI demonstrating diffuse leptomeningeal enhancement along the surface of the cord, suggestive of an infectious or inflammatory process; axial T2 Flair postcontrast MRI brain (C) demonstrating diffuse leptomeningeal and pachymeningeal enhancement.

Empiric intravenous vancomycin, ceftriaxone, and metronidazole were initiated immediately. Lumbar puncture yielded seropurulent CSF with marked neutrophilic pleocytosis, markedly elevated protein concentration, and reduced glucose. The patient underwent emergent irrigation and debridement of the lumbar surgical site, where copious purulent material was encountered deep to the fascia. Careful exploration of the dura, including multiple Valsalva maneuvers, revealed no evidence of a CSF leak or dural defect. The wound was thoroughly debrided and irrigated with 3 liters of normal saline with cefazolin. He returned to the intensive care unit (ICU) intubated and in critical but stable condition.

Both operative and CSF cultures grew MRSA, and antimicrobial therapy was narrowed to intravenous vancomycin. His hospitalization was further complicated by persistent abnormal movements with hiccup-like activity characterized as diffuse encephalopathy, which required prolonged ventilatory support and seizure prophylaxis. Following multidisciplinary management involving orthopedic surgery, infectious disease, neurology, and critical care, the patient demonstrated gradual neurological improvement. He was extubated on postoperative Day 12 and transferred to the general floor on postoperative Day 14 with a peripherally inserted central catheter for long-term antibiotic therapy. He completed a 6-week course of intravenous vancomycin and was discharged to inpatient rehabilitation on postoperative Day 27.

The patient was discharged to a subacute rehabilitation facility ~3 months after his initial admission, and at that time was alert and oriented to person, place, and time, with 5/5 strength in the bilateral upper and 4/5 strength in the bilateral lower extremities. Mild cognitive impairment persisted, characterized by moderate deficits in memory, comprehension, and problem-solving.

At 6-month follow-up, he had returned home, resumed part-time work as a jeweler, and was participating in home physical therapy. He ambulated independently without assistive devices and demonstrated full strength in all extremities, with only mild residual cognitive deficits.

## Discussion

Healthcare-associated meningitis following lumbar spine surgery is an uncommon but potentially life-threatening complication. Most reported cases occur in the setting of incidental durotomy or persistent postoperative CSF leakage, allowing contamination of the subarachnoid space [3, 4, 7]. In contrast, no CSF leak or dural defect was identified during either the revision microdiscectomy or subsequent irrigation and debridement in the present case despite meticulous exploration. Although an occult dural injury that sealed prior to re-exploration cannot be excluded, contiguous spread from a deep surgical site infection represents another plausible mechanism [8, 9]. This case demonstrates that postoperative meningitis may occur even in the absence of a recognized dural violation.

Diagnosis can be challenging because the early clinical manifestations of postoperative meningitis often overlap with expected postoperative findings or medication-related effects. Progressive alteration in mental status, persistent fever, seizures, severe headache, neck stiffness, or new neurological deficits should prompt immediate evaluation for central nervous system infection. Although postoperative inflammation may complicate interpretation of CSF findings, the diagnosis should be based on the overall clinical presentation in conjunction with neuroimaging, microbiologic testing, and CSF analysis, as no single CSF parameter reliably distinguishes postoperative bacterial meningitis from aseptic postoperative inflammation [10, 11]. In our patient, diffuse leptomeningeal enhancement on MRI together with profound CSF neutrophilic pleocytosis, markedly elevated CSF protein concentration, and positive CSF cultures growing MRSA established the diagnosis.

The microbiology of healthcare-associated meningitis differs from that of community-acquired disease, with *S. aureus*, coagulase-negative staphylococci, and aerobic gram-negative bacilli representing the predominant pathogens [1]. Current guidelines recommend prompt empiric therapy with vancomycin combined with an antipseudomonal β-lactam until culture and susceptibility results become available, after which antimicrobial therapy should be tailored accordingly [1]. In this case, early surgical debridement, prompt initiation of broad-spectrum antibiotics, subsequent culture-directed vancomycin therapy, and multidisciplinary critical care resulted in substantial neurological recovery despite an initially fulminant presentation.

This case highlights several important clinical lessons. MRSA meningitis may occur following revision lumbar microdiscectomy despite the absence of an identifiable dural tear. Progressive neurologic decline in the early postoperative period should prompt immediate evaluation for meningitis, even when alternative explanations such as medication effects appear plausible. Early recognition, CSF analysis, surgical source control, and targeted antimicrobial therapy remain essential for optimizing neurological recovery in this rare but potentially devastating complication.
